# Evaluation of Scaling Invariance Embedded in Short Time Series

**DOI:** 10.1371/journal.pone.0116128

**Published:** 2014-12-30

**Authors:** Xue Pan, Lei Hou, Mutua Stephen, Huijie Yang, Chenping Zhu

**Affiliations:** 1 Business School, University of Shanghai for Science and Technology, Shanghai, China; 2 Computer Science Department, Masinde Muliro University of Science and Technology, Kakamega, Kenya; 3 College of Science, Nanjing University of Aeronautics and Astronautics, Nanjing, China; Tianjin University, China

## Abstract

Scaling invariance of time series has been making great contributions in diverse research fields. But how to evaluate scaling exponent from a real-world series is still an open problem. Finite length of time series may induce unacceptable fluctuation and bias to statistical quantities and consequent invalidation of currently used standard methods. In this paper a new concept called correlation-dependent balanced estimation of diffusion entropy is developed to evaluate scale-invariance in very short time series with length 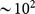. Calculations with specified Hurst exponent values of 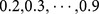 show that by using the standard central moving average de-trending procedure this method can evaluate the scaling exponents for short time series with ignorable bias (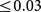) and sharp confidential interval (standard deviation 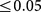). Considering the stride series from ten volunteers along an approximate oval path of a specified length, we observe that though the averages and deviations of scaling exponents are close, their evolutionary behaviors display rich patterns. It has potential use in analyzing physiological signals, detecting early warning signals, and so on. As an emphasis, the our core contribution is that by means of the proposed method one can estimate precisely shannon entropy from limited records.

## Introduction

A stochastic process behaves scale-invariance if the probability distribution function (PDF) of its displacements 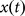 obeys,
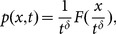
(1)where 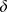 is the scaling exponent. Ordinary statistical mechanics is intimately related to the Central Limit Theorem [1], which implies the Gaussian form of the function 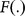 with 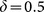 [2]. The scaling exponent tells us quantitative deviation of a phenomena from ordinary mechanics, displays its real physical nature. Scale-invariance has been making great contributions to progresses in diverse research fields [3], such as establishment of fractal market hypothesis [4], evaluation of healthy states from physiological signals [5], and identification of genes encoding proteins in DNA sequences [6]–[9]. But how to evaluate exactly the values of 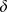 from real world time-series is still an open problem.

Variance-based methods, e.g., wavelet analysis [10], [11] and de-trended fluctuation analysis (DFA) [12]–[16], employed in literature as standard tools, require an assumption, namely, 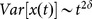. It is valid for Brownian motions, but for Levy walks we have 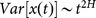 with 
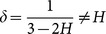
 [17]. Scale-invariance in Levy flights can not be detected qualitatively at all due to divergence of the second moment of displacements.

A successful effort in developing complementary methods is the diffusion entropy analysis (DE) [17]–[19] proposed by Scafetta et. al.. From a stationary time series, one can extract all the possible segments with a specified length. Regarding the length of the segments as duration time, each segment is mapped to a realization of a stochastic process, namely, a trajectory starting from the original point. All the realizations form an ensemble, which can be described by a diffusion process. If Eq.(1) stands for the PDF of displacement of the ensemble, a simple computation shows that there exists a linear relation between Shannon entropy, called diffusion entropy, and the logarithm of segment length, slope of which equals to 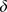. This entropy-based method attracts extensive attentions (see, for examples, [20]–[25]) for two reasons. It is dynamical process independent, namely, it can give simultaneously reliable values of scaling exponents for fractional Brownian motions and Levy processes. What is more, by comparing its result with that of variance-based methods, one can identify from time series the underlying dynamical mechanisms (Brownian motion or Levy process).

A key challenge in practice is that finite length of real-world time series may reduce the accuracy of the estimation of fractal exponents. Real-world time series are generally very short. Sometimes, a long record is available, but phase transitions may occur in the monitoring duration. To identify different behaviors of the complicated system, we should separate the long time series into short segments. Specially, at present time, researchers' attentions are moving to specific characteristics in each sample, instead of the common characteristics existing in many samples. Hence, a tool should have good performance for single and short time series. Statistically, a high-confidential estimation of scaling exponent means ignorable bias and sharp confidential interval. Our goal in this paper is to improve the initial diffusion entropy concept to a high-performance version to evaluate scaling behaviors embedded in single and short (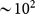) series.

Argument on the finite length effects has been persisting for decades. To cite an example, detailed calculations by A. Eke, et.al. [26]–[31] propose that one needs series of at least 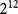 data points to get reliable results. On the contrary, in the paper by D. Delignieresb [32], by integrating different methods into a complicated flowchart, the authors show that the loss of accuracy of the estimation in short time series (at least 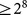) is not as dramatic as expected. However, this conclusion is based upon a procedure of statistical average over 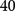 realizations, which requires a total of 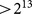 records.

Recently, by minimizing the summation of statistical error and bias, Bonachela et al. [33], [34] proposed a balanced estimation of Shannon entropy for a small set of data, which performs well even when a data set contains few tens of records. Replacing the original Shannon entropy with the balanced entropy estimation, we convert the DE method to a new version, called balanced estimation of diffusion entropy (BEDE) [35], [36]. Detailed calculations on constructed fractional Brownian series, stock market records, and physiological signals show that the BEDE is a possible way to evaluate scaling behaviors embedded in a single and short time series with several hundreds length.

The BEDE method proves it powerful, but there are still several essential questions to be answered. First, in the deduction of the original balanced estimator of entropy, the correlations between elements in different bins are simply neglected. Actually, the summation of the elements in all the bins should be a constant, i.e., the total number of constructed realizations. Is this simple assumption proper or not? Second, for long time series, effect of de-trending procedure can be ignored. But for very short time series, the effect may lead to serious mistakes. How the technical details in de-trending procedure affect the results? Third, and the most important for applications, what a performance (bias and confidential interval) can be reached when we considering a single sample with 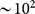 length?

In the present work, we give clear answers to the above questions. Our contribution is threefold:

First, we consider the correlations between elements in all the bins. It turns out to be a key step to increase significantly accuracy of estimation of entropy when the number of bins tend to large. Accordingly, we present a new estimation of the total entropy, called correlation-dependent balanced estimation of diffusion entropy (cBEDE). By using cBEDE one can estimate precisely Shannon entropy from limited samples, which is a serious challenge in diverse research fields. This is the key contribution.

Second, in the methods of cBEDE and BEDE, there exists a null hypotheses that if we re-scaled at each duration time 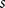 the displacements by the way of 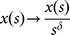, the resulting estimations of entropy are independent with 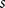. We test this assumption and accordingly introduce a modification to BEDE and cBEDE.

Third, BEDE and cBEDE are valid only for stationary time series. In literature, several de-trending procedures are proposed, such as the polynomial fit [12]–[16] and the central moving average [37]–[42]. In the present paper we investigate the performances of BEDE and cBEDE by using the standard central moving average (SCMA) solution and its mutation. It is found that the SCMA makes the cBEDE works best.

The three contributions lead to a high performance of cBEDE. For a single short time series with 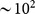 length, by using the stadard SCMA procedure cBEDE can estimate its scaling exponent with ignorable bias (less than 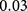) and significantly high confidence (standard deviation less than 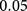). On the contrary, the confidential interval for the BEDE method is about 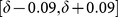 for the both de-trending methods, covering about an interval of about 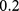.

As an example, application of this method to walks, we find rich patterns in the evolutionary behaviors of scaling invariance embedded in the stride series.

## Method and Materials

### Method

#### A Brief Review Of Diffusion Entropy [17]

Let us consider a stationary time series, 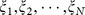. All the possible segments with length 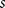 read,



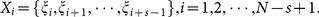
(2)


Now we regard 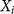 as a realization of a stochastic process, namely, a trajectory of a particle starting from the original point and the duration time is a total of 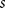 time units. All the 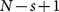 trajectories form an ensemble, whose displacements, 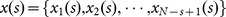, are,



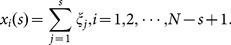
(3)


Let us find the distribution region of the displacements 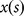, namely, 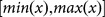, and divide it into 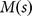 bins with the same size, 
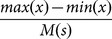
, each. The PDF can be naively approximated as,



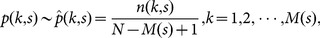
(4)where 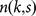 is the number of displacements occurring in the *k*th bin. The consequent naive estimation of diffusion entropy of the process reads,



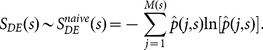
(5)


We assume the time series behaves scale-invariance, namely, 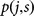 satisfies,



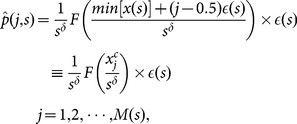
(6)where 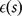 is the window size, and 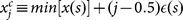, i.e., the central point of the *j*th bin. Eq.(5) can be rewritten as,



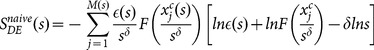
(7)


If the length of the time series is infinite, i.e., 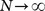 and 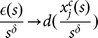, the naive estimation of entropy can be approximated with a integral form, which reads,



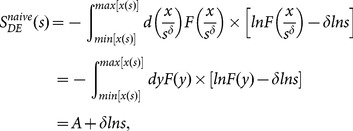
(8)where 
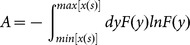
, a constant.

Hence, the simple relation of Eq. (8) can be used to detect scalings in time series. It is the first tool yielding correct scalings in both the Gaussian and the Lévy statistics. For this reason, it is used to detect scale-invariance in diverse research fields [43], such as solar activities [44]–[48], spectra of complex networks [49], physiological signals [50]–[54], DNA sequences [55], [56], geographical phenomena [57]–[59], and finance [51], [60].

#### De-trend Procedure

A real-world time series is generally non-stationary. In literature several novel solutions are proposed to subtract trends in time series, such as the polynomial fit [12]–[16] and moving average [37]–[42] in DFA method. In the present work we adopt the central moving average scheme. From a real-world time series, 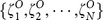, one can calculate the trend series, whose elements are,
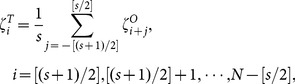
(9)where [.] is the integral function, and 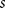 is identical with the duration time in Eq.(8). The consequent de-trended time series can be calculated as,



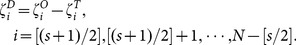
(10)


The resulting series is regarded as stationary. This procedure is called standard central moving average scheme (SCMA).

As comparison, we adopt also a mutation of SCMA. In calculations, if the standard central moving average is used, the length of the resulting time series is 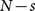, from which one can extract a total of 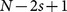 segments to estimate probability distribution function. The loss of 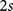 records maybe neglected if time series is long enough, but for short time series the lost records are valuable. To take into account of contributions of the lost records, a mutated solution is to loose the procedure of SCMA in the two end parts of time series, namely, the elements of trend read,



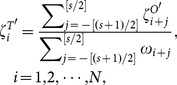
(11)where, for 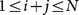, 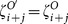 and 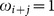, otherwise, 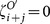 and 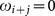. And the de-trended time series reads,



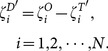
(12)


The standard central moving average is conducted strictly only in the cental part of the series. This method is denoted with lSCMA in this paper.

#### Correlation-Dependent Balanced Estimation of Diffusion Entropy

In the DE method, the bin size 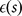 is generally chosen to be a certain fraction of the standard deviation of the considered time series. With the increase of 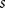, the characteristic distribution width of 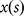 (i.e., standard deviation of 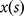) extends rapidly according to 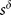, and the number of bins, 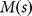, will increase in a speedy way. For finite 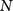, the naive estimation of relative frequencies may lead to large fluctuations and bias to the calculations in downstream steps. Defining an error variable, 
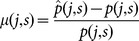
, a straightforward computation leads to a rough estimation of bias, 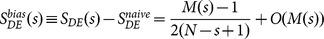 [33]. Consequently, 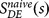 deviates significantly from the true entropy not only statistically but also systematically.

Our goal is to find a proper estimation of diffusion entropy to reduce simultaneously the bias and the variance as possible, which can be formulated as an optimal problem [34]. For simplicity, the variable 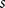 is not written explicitly in the following formula. Let us denote the occurring probabilities and realization numbers in the 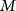 bins with 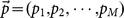, and 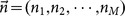, respectively. One can define bias and statistical fluctuation as,



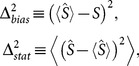
(13)where 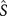 is the estimation of real diffusion entropy 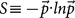, and 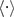 the average over all possible configurations of 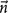. To balance the errors, we consider the total error averaged over all the configurations of 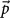, which reads,
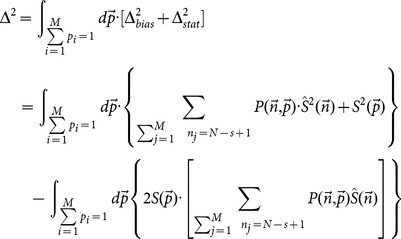
(14)where 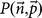 is the binomial distribution,



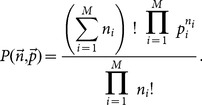
(15)


The expected values of 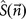 should lead to the minima of the averaged error, which requires a necessary condition reads,



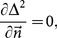
(16)for all the possible configurations of 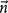. A simple algebra leads to,



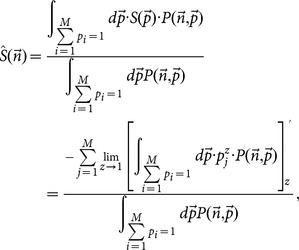
(17)where we use the identify, 
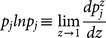
.

After a very cumbersome computation (see Appendix), we deduce the final estimation of diffusion entropy, which reads,



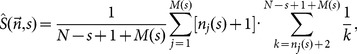
(18)called correlation-dependent balanced estimation of diffusion entropy (cBEDE). One can find that for the specific case of 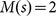, cBEDE degenerates to the BEDE. However, our calculations show that when 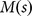 is large, there exists great difference between them.

#### Null-hypothesis-based correction

From the scale-invariance definition one can find that the characteristic width of displacement distribution increases according to 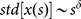. For each duration time, 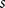, we consider re-scaled displacements, which read,



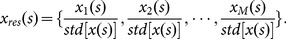
(19)


Behaviors of entropy estimations for the re-scaled displacements 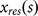 should be independent with duration time 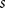. This hypothesis can be used to test and correct proposed methods. Denoting entropy estimations for original and re-scaled displacements with 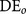, 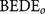, 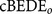 and 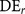, 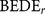, 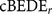, respectively, the final calculated entropy estimations read,



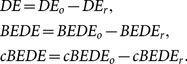
(20)


### Materials

#### Fractional Brownian Motions

Fractional Brownian motions [61], [62] are used to evaluate and compare the performances of DE, BEDE, and cBEDE. A fBm refers to a continuous-time Gaussian process whose characteristics depends on its Hurst exponent 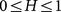. It is scale-invariance, namely, the PDF of its increment 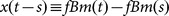 satisfies 
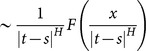
. It has also a convergent variance of increment obeys a power-law, 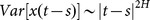. In this work, the built-in program 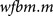 in 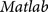
® is used to generate the fBm series.

#### Stride Series

The empirical data are the stride series of a total of 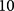 young healthy volunteers [63], denoted with 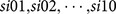, respectively. The participants have not historical records of any neuromuscular, respiratory, and/or cardiovascular disorders, and are not taking any medication. The ages distribute in a range of 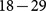 year, the average of which is 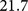 year. The height and weight center at 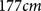 and 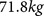, with standard deviations 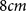 and 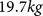, respectively. All the objects walk continuously around an obstacle-free (approximately oval path) on ground level measuring 225m or 400m in length. The stride interval is measured by using an ultrathin, force-sensitive switch taped inside one shoe. Each object walks four trials, i.e., slow, normal, fast, and metronome-regulated. Slow, normal, fast walks indicate that the corresponding mean stride intervals are 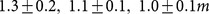 and 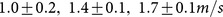, respectively. The lengths of the stride time series distribute from 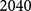 to 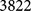 steps.

## Results

### Performance of cBEDE

**Figure 1:**
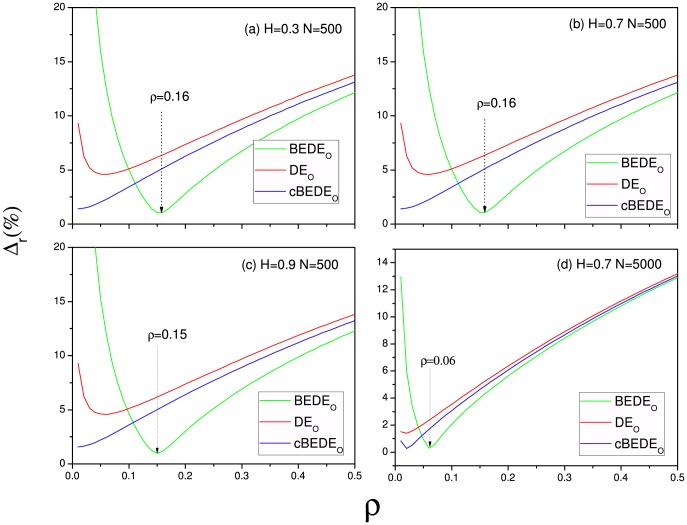
Relative errors of DE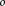, BEDE 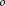 and cBEDE 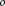 versus bin size 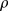. (a) H = 0.3, N = 500. (b)H = 0.7, n = 500.(c)H = 0.9, N = 500.(d)H = 0.7, N = 5000. Each curve is an average over 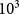 realizations. When 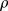 is large, DE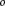, BEDE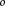, and cBEDE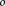 are very close. In the displayed range of 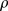, cBEDE decreases monotonically, while BEDE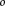 decreases to a minima and then increases rapidly to unacceptable values.

Fig. 1 presents several typical examples to illustrate performances of 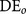, 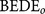, and 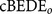 when the number of bins changes. For each Hurst exponent value 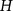, we generate 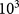 independent fBm series. The window size is chosen to be 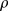 times that of the standard deviations, while the duration time keeps to be a constant, 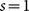. The smaller the value of 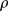, the larger the number of bins the displacement region is being divided into. We calculate the bias 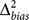 and the statistical fluctuation 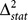. The relative error is defined as, 
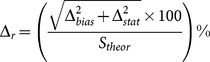
, where 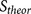 is the corresponding theoretical value of entropy.

With the decrease of 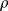, the relative error of 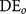 decreases rapidly and reaches a minima at a small value of 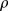. The 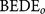 coincides best with the theoretical values of entropy when the window size is large, but when the window size becomes small, i.e., the bin number tends large, its deviation increases sharply to unacceptable values. One can find that 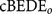 has always smaller deviation rather than 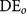 does, especially in the region of small values of 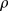. In the considered region of 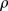 the relative error of 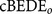 decreases monotonically. For the cases of 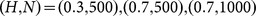, and 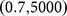 (as shown in Fig. 1(a)-(d)), the values of 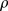 corresponding to the minima of 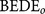 are 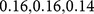, and 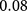, respectively. In the procedure of 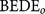, the bin number increases according to 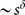. The corresponding values of 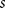 are 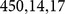 and 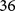, respectively. To obtain a reliable scaling exponent requires the scaling range being large as possible, namely, the larger the bin number the better. Hence, we can expect a best performance of 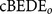.

The relative error is determined by two factors, namely, number of realizations, 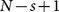, and number of bins, 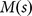, the displacement interval being divided into. With the increase of 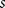, 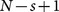 decreases while 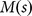 increases rapidly according to 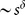.

At the beginning (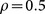), the occurring numbers in the bins are large enough, and the finite effect can be neglected. With the decrease of 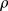 (increase of bin number), much more details in the probability distribution function (PDF) can be captured, which leads to decreases of relative errors for cBEDEo, BEDEo, and DEo.

At the same time, increase of bin number will lead decrease of occurring numbers in the bins, which means increase of bias and fluctuations due to finite occurring numbers. By considering the constraint of the total realizations being constant, error of 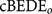 decreases monotonically. While there occur transition points for the errors of 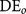 and 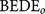. The improvement from BEDE to cBEDE is a necessary step.

But when 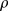 becomes small, the occurring numbers in the bins are not large, and the finite effect tends to dominate the relative errors. For the cBEDEo, the consideration of the total number of realizations being constant guarantees the precision of estimations. Consequently, in the considered range of 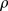 the relative error can decrease monotonically. While the estimation errors for BEDEo and DEo will increase significantly. The minimum values of DEo and BEDEo occur.

To obtain reliable scaling behavior, the considered range of 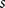 should be large as possible. Hence, how to guarantee a correct estimation of diffusion entropy at large 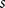 (i.e, small values of 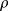) is the key problem. The significant precision of cBEDE at small 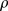 makes it possible to evaluate scaling exponent from large range of 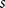. Hence, the high estimation precision of cBEDE at small values of 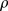 is important.

**Figure 2:**
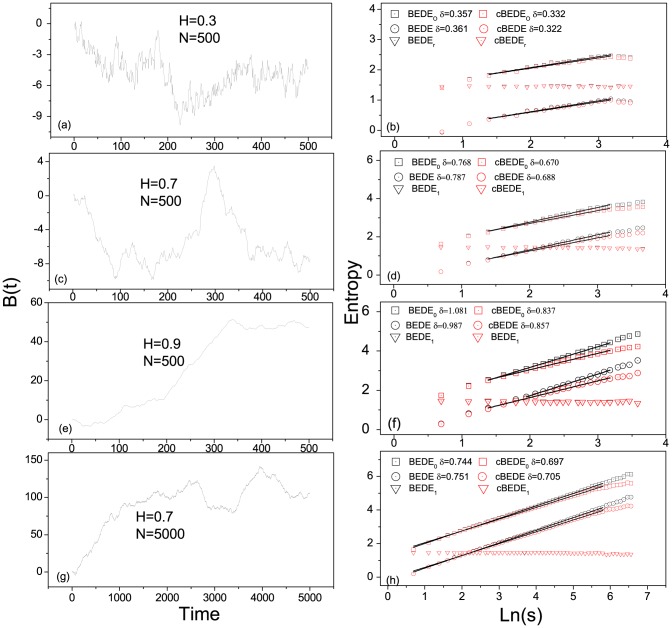
Several typical examples of entropy estimation by means of cBEDE and BEDE. The SCMA de-trending scheme is employed. Panels (a),(c),(e) and (g) are generated fBm time series with 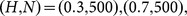 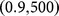 and 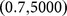, respectively. Panels (b),(d),(f) and (h) are the corresponding entropy estimations of cBEDE and BEDE. Slopes for re-scaled time series are small minus values, do not vanish even for the case of 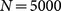 in (h). cBEDE provides correct estimations of 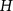, while BEDE overestimates 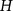 up to about 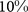.

By using the SCMA de-trending scheme, Fig. 2 provides several examples of entropy estimations versus duration time 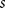. One can find that the entropies for re-scaled series, 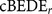 and 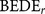, obey straight lines with small minus slopes, whose absolute values are less than 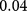. The slope does not vanish even when the length 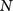 becomes 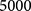 in Fig. 2(g–h). Hence, this bias comes from the specific methods, which should be corrected in the procedure of detecting scale-invariance.

For the case of 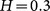 which is less than 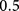, as shown in Fig. 2(a–b), there is not distinguishable differences between the curves of cBEDE and BEDE. While for 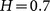 and 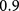 with 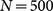 (see Fig. 2(c–d) and Fig. 2(e–f)), in the range of small duration time 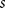, the curves of 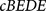 and 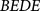 are almost undistinguishable. When 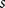 becomes large enough, the curves of BEDE increase in a speedy way compared with that of cBEDE, though they all obey the relation 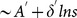 in much large ranges of 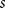. For 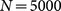, as an example for series with enough length, one can find only slight difference between cBEDE and BEDE in a considerable wide range of 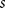. These findings are verified by a large amount of calculations for fBm series with different values of 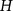 and 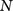.

Herein, we propose an algorithm to estimate the scaling exponent in wide interval of 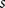 as possible. From a total of 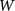 values of entropy estimations, we select initially a range of points, 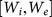, where the relation 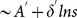 stands with a high precision. At each step we extend the range to include more values of entropy estimations and calculate the value of 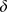. Let us denote values of 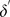 for two successive steps with 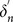 and 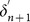, respectively. The procedure iterates until a criterion is broken through. The criterion is twofold. The difference between two successive values of 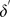 is less than a criterion 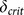, namely, 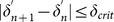. And the aggregation of differences for all the steps should be limited to a certain degree, namely, 
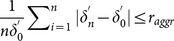
. By this way we can find the largest range of 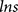, in which the scaling exponent can be estimated correctly.

In calculations we set 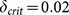, and 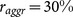. The values of 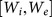 depend on de-trending procedures, i.e., equal to 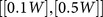 for the SCMA, 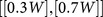 for the lSCMA. In Fig. 2, for the cases of 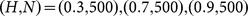 and 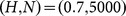, the resulting slopes of BEDE and cBEDE are 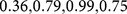, and 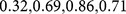, respectively. The BEDE gives unacceptable large values of 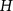 (overestimated about 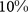), while the slopes of cBEDE are very close to the expected values.

**Figure 3:**
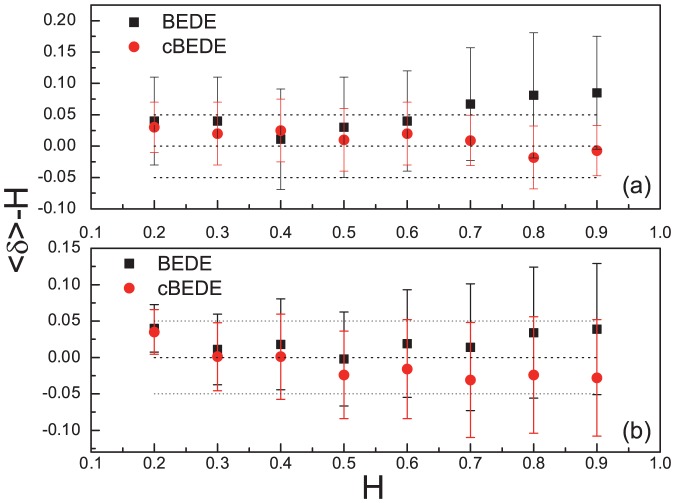
Bias and fluctuation of estimated scaling exponents by means of cBEDE and BEDE. For each Hurst exponent, statistical average and fluctuation are obtained over an ensemble of 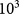 independent realizations with length 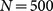. (a)–(b) SCMA and lSCMA de-trending procedures are employed, respectively. For SCMA de-trending procedure, cBEDE can evaluate scaling exponents with small bias (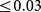) and standard deviation (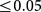).

These findings are confirmed statistically in Fig. 3, in which we present a comparison between the two solutions of de-trending procedure. The average and standard deviation of estimated scaling exponents are obtained over 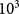 independent realizations (with length 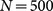) for each specific value of 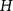. For the de-trending procedure SCMA, as shown in Fig. 3(a), the cBEDE can estimate 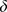 with acceptable small values of bias (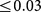) and standard deviation (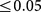), while for the BEDE the bias and standard deviation can reach 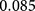 and 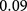, respectively. For the lSCMA procedure (shown in Fig. 3(b)) cBEDE can estimate 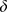 with bias less than 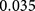 and standard deviation less than 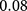, which are almost the same with that the BEDE performs, i.e., the bias less than 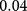 and the standard deviation less than 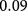. Hence, by using the SCMA procedure, the cBEDE has significantly high performance, namely, in the wide range of 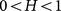 it can estimate scaling exponents with ignorable bias and significantly sharp confidential interval.

The positive bias for BEDE in Fig. 3(a) and Fig. 3(b) is consistent with the results in Fig. 1 and Fig. 2. One can find that the BEDE overestimates diffusion entropy when window size becomes large and accordingly the scaling exponents up to 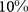. While the cBEDE can give precise estimation of entropy when the window size becomes large.

The performance of SCMA is better than that of lSCMA. The reason may be that our method can depress efficiently the finite length induced fluctuations and bias of estimated entropy. Accordingly, the lost of data at the start and the end in the SCMA does not lead to serious errors. While in the lSCMA the looseness of standard central moving average at the end and start leads to serious errors to the cBEDE method.

As a summary, to evaluate reliably scaling exponents require a joint consideration of effects from three factors, namely, finite length, de-trending procedure, and null-hypothesis.

### Scaling Behaviors For Stride Series

**Figure 4:**
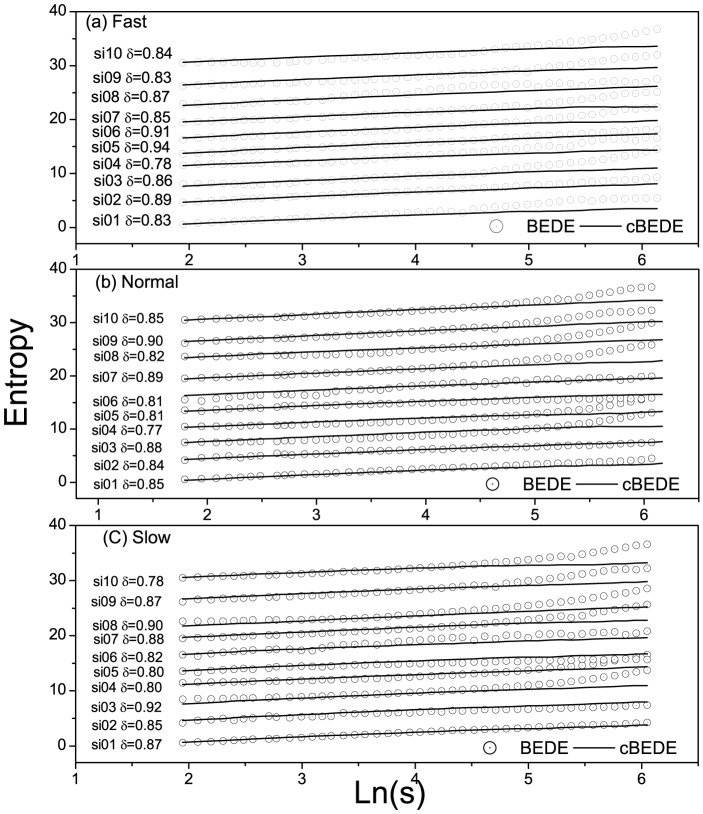
Scaling-behaviors of stride time series by using BEDE and cBEDE. SCMA de-trending scheme is used. (a)-(c) correspond to normal, slow, and fast walking trials, respectively. cBEDE and BEDE are illustrated with solid lines and gray symbols, respectively. The lengths of the stride time series distribute from 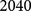 to 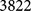 steps.

By using the SCMA de-trending procedure, we calculate cBEDE versus 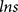 for all the stride series. As shown in Fig. 4, the cBEDE curves (solid lines) are all straight lines (ignorable slight bending downward when 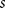 becomes large), namely, the time series behave almost perfect scale-invariance. For comparison we present also the BEDE curves (gray symbols), which bend upward when 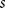 becomes large. Consequently, cBEDE can evaluate precisely the scaling exponents, while BEDE will over-estimate the values of scaling exponents.

The scaling exponents for fast, normal, and slow (as shown in Fig. 4(a)–(c)) distribute in the range of [0.78, 0.94], [0.77, 0.90], and [0.78, 0.92], respectively. One can find that for each subject there exist not significant differences between the scaling exponents for different walking rates, except the subject numbered 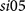, whose scaling exponent is 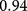 for the fast series which is significantly larger than that for normal and slow series (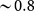).

**Figure 5:**
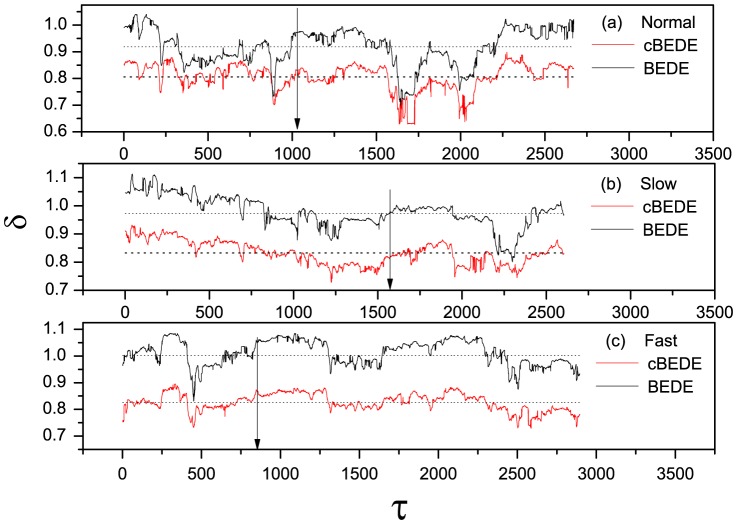
Evolution of scaling behavior for the subject numbered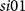 by using BEDE (red line) and cBEDE (black line). (a)–(c) correspond to fast, normal, and slow trials, respectively. Let a window with length 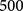 slide along the original time series. Scaling exponent for the covered segment is used to represent the corresponding local behavior. There exist rich sub-structures in the walking durations. The cBEDE and BEDE curves at the points marked with arrows will be shown in Fig. 6.

During the experiments we assume the physiological states of the volunteers remain unchanged. Let a window slide along the original series. At the *τ*th step, the window covers the segment 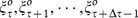, where 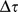 is the size of the window. Scaling exponent for the covered segment can be used to represent the local scaling behavior at time 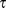. Calculations show that the behavior of scaling exponent changes with time significantly, namely, there exist rich fine structures in the walking durations. As a typical example, we show in Fig. 5 the evolutionary behavior of scaling exponent for the subject 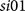. The window size is selected to be 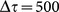. The BEDE over-estimate the values of scaling exponents.

**Figure 6:**
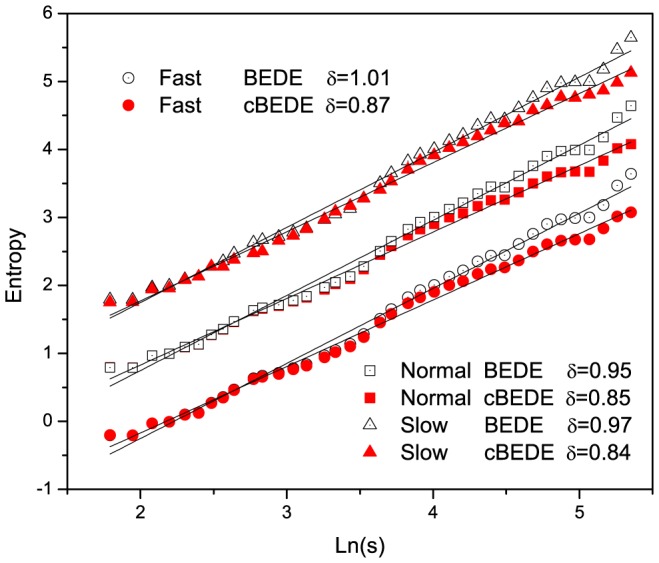
Local scaling behaviors corresponding to the points in Fig. 5 marked with arrows. The over-estimation of BEDE is due to the bending upward when 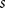 becomes large.

To show how the BEDE overestimates the value of scaling exponent, we present in Fig. 6 the BEDE and cBEDE curves for the three specific segments marked in Fig. 5 with the arrows. One can find that the curves for cBEDE are almost straight lines, while that for BEDE bend significantly upward (i.e., being overestimated).

**Figure 7:**
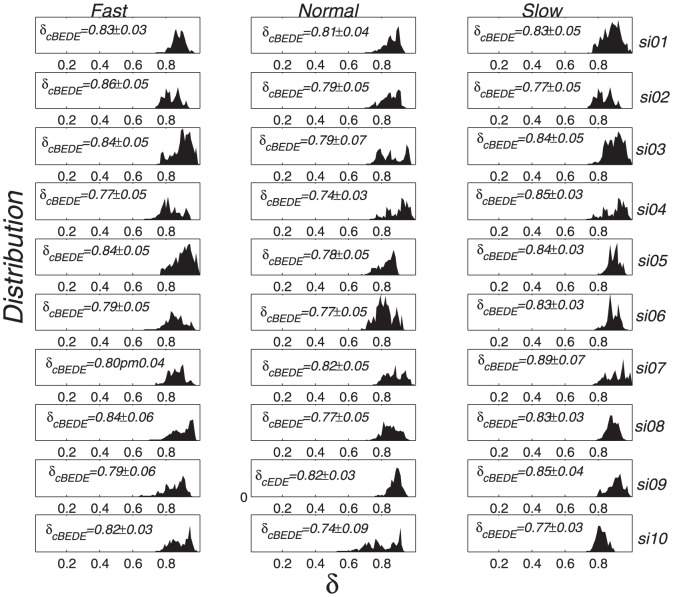
Distributions of local scaling exponents for each subject.

Fig. 7 shows the distributions of local scaling exponents for each subject. One can find that the shapes of distribution are completely different, though there exist little differences between the averages and standard deviations.

The rich patterns in the curve of scaling exponent evolution and scaling exponent distributions show us that in the walking duration the persistence of physiological state changes significantly. But a conclusive physical discussion requires a detailed investigation based upon enough experimental records, which are invalid at present time. As a suggestion we hope the forthcoming experiments can monitor simultaneously multi-parameters of physiological state, such as stride, breathing, and heartbeat.

## Conclusion and Discussion

In summary, scaling invariance holds in a large number of complex systems and has been making great contributions in diverse research fields. Some powerful algorithms have been developed in literature as standard tools to calculate scaling exponents in time series. But how to evaluate scaling behaviors embedded in very short time series (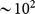 length) is still an open problem.

In this paper, we propose a new concept called correlation-dependent balanced estimation of diffusion entropy (cBEDE) to evaluate scaling invariance embedded in short time series. Contribution in this work is threefold. Theoretically, the correlations between occurring numbers in different bins are considered, which leads to a much more exact estimation of diffusion entropy, as supported by a large amount of numerical results. By re-scaling displacements at each duration time 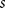, the specific method related bias is also corrected. The performance of the proposed method is evaluated by using central moving average de-trending procedure (SCMA) and its mutation (lSCMA).

Calculations with specified values of Hurst exponent (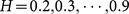) show that for short time series with 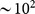 length, by using the SCMA procedure cBEDE can estimate scaling exponents with ignorable bias (less than 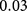) and significantly high confidence (standard deviation less than 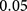). Comparison shows that taking account of the correlations between elements in all the bins is the key step for us to have the so good performance.

As an example, application of this method to walks finds rich patterns in the evolutionary behaviors of scaling invariance embedded in the stride series. In the experiments, we try to keep the condition unchanged. By this way, one hope the states of volunteers keep the same, as being assumed in literature. But our works show that in the duration of walk, the state of a volunteer may change significantly.

It should be noted that scaling behaviors embedded in short time series is just a typical example of the potential applications of cBEDE. The core contribution herein is a new method that can estimate Shannon entropy with high performance from limited records.

Very recently, reconstructing relation networks from mono/multi-variate time series attracts special attentions for its powerful in distinguishing time series generated by different dynamical mechanisms. To cite examples, Zhang et al. [64], [65] for the first time propose a method to map a time series to network, in which the time series is separated into segments according to pseudo-periods. The segments with strong cross-correlations are linked. While in the recurrence plot [66]–[75] a mono/multi-variate time series is divided into equal-sized segments by using the phase-space reconstructing technique. Then the segments are networked according to the correlation strengths between them. In the methods, the key problem is how to extract from short time series (segments) reliable relations. We hope the concept of cBEDE can make significant contributions in this topic.

First, it can be used to extract state information from limited records. Very recently, by using the cBEDE we report for the first time the long-term persistence embedded in rating series in online movie systems [76]. The characteristic length of the series are 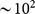, which makes the other methods invalid. The findings provide a new criterion for theoretical models, and provide us some knowledge on how collective behavior of an online society is formed from individual's behaviors.

Second, it can be used to extract evolutionary behaviors from one-dimensional time series. Here we cite several interesting problems. Detection of early warning signals [77] attracts special attentions for its special application in prediction of disasters, which requires an estimation of a complex system's state with considerable high precision from short time series. Diagnosis of disease [78] needs also a valuable evaluation of healthy state and its evolutionary behavior from limited records. To find mechanism embedded in financial records, we should know the scaling behavior of a stock market from a second to a day, a month, or even a year time-scale. When the sampling interval is large, the available time series will shrink to a limited length.

Third, it must be used when we address multivariate time series. To cite an example, a complicated system contains many networked elements, relationships between which can describe quantitatively the global state of the system [79]. Monitoring dynamical process of the system generates a multivariate time series. Shannon entropy based concepts, such as mutual entropy [80], [81] and transfer entropy [82], multi-scale cross entropy [83] are proposed in literature to reconstruct the relationship network between the elements from the produced time series. One should divide the distribution region of a bivariate series into some rectangles, and reckon the occurring numbers of samples in each rectangle. If each variate interval is divided into 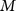 bins, the resulting number of rectangles will be 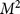, which makes the finite length problem a serious challenge.

## Appendix

The estimations of entropy read, 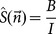, where



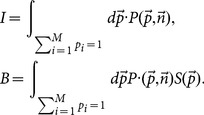
(A.1)


### Analytical expression of 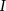


Let 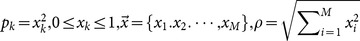, we have,
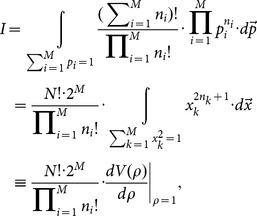
(A.2)where,
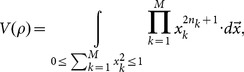
(A.3)and 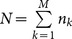.

With the help of the spherical coordinate expressions of 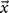,
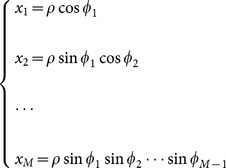
(A.4)a simple computation leads to,
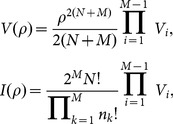
(A.5)where
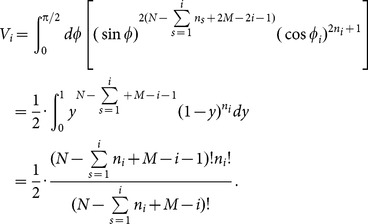
(A.6)


Hence, we have the analytical expression of 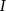,
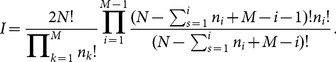
(A.7)


### Analytical expression of 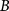


Using the identify of 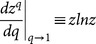, analogous procedure leads to,



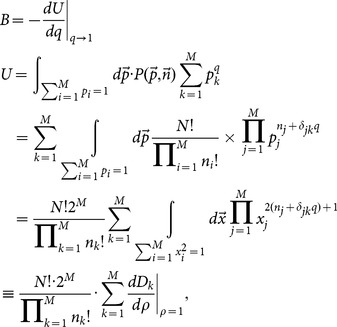
(A.8)where,
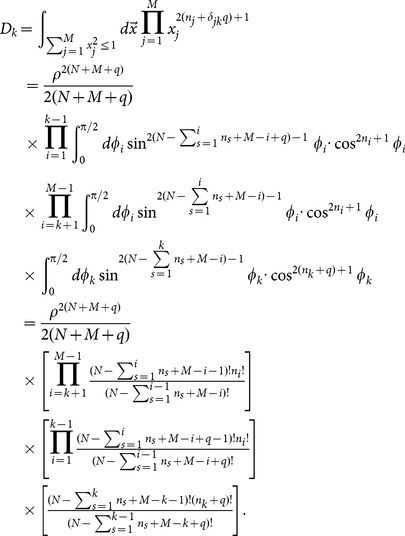
(A.9)


The analytical expression of 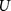 reads,



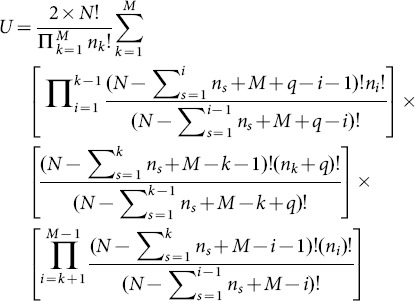
(A.10)


The final explicit expression of 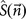 reads,



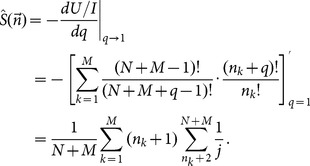
(A.11)


In the present paper, at the duration time 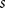 the ensemble contains 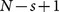 trajectories, so the correlation-dependent balanced estimation of diffusion entropy reads,



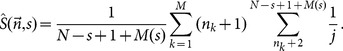
(A.12)


When we neglect correlations between occurring numbers in different bins, one can simply reduce the distribution 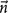 into two components, namely, the occurring number in the considered bin and the total number of particles occurring in other bins. The consequent value of 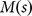 is 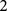. In this case cBEDE degenerates to BEDE.
